# Thoracic Aortic Aneurysm and Factors Affecting Aortic Dissection

**DOI:** 10.3390/jpm10040153

**Published:** 2020-10-02

**Authors:** Petr V. Chumachenko, Anton Yu. Postnov, Alexandra G. Ivanova, Olga I. Afanasieva, Maksim A. Afanasiev, Mariam Bagheri Ekta, Vasily N. Sukhorukov, Grigoriy I. Kheimets, Igor A. Sobenin

**Affiliations:** 1 Laboratory of Medical Genetics, National Medical Research Center of Cardiology, Institute of Experimental Cardiology, 15-a 3-rd Cherepkovskaya Str., 121552 Moscow, Russia; chumach7234@mail.ru (P.V.C.); afanasieva.cardio@yandex.ru (O.I.A.); am-mma@mail.ru (M.A.A.); gregorykheimets@rambler.ru (G.I.K.); igor.sobenin@gmail.com (I.A.S.); 2 Research Institute of Human Morphology, Laboratory of Cellular and Molecular Pathology of Cardiovascular System, 3 Tsyurupy Str., 117418 Moscow, Russia; ms.bvgheri@gmail.com (M.B.E.); vnsukhorukov@gmail.com (V.N.S.); 3 Russian Research Center of Surgery, Lane Abrikosovsky, 2, 119991 Moscow, Russia; gussona@gmail.com

**Keywords:** thoracic aortic aneurysm, dissection, medianecroses, vasa vasorum, inflammation, lipoprotein(a)

## Abstract

This study is aimed at investigating the relationship between inflammation, the number of vasa vasorum, and the presence of lipoprotein (a) [Lp(a)] in the aortic aneurysm wall, as well as the relationships of these pathological processes with the development of aneurysm wall dissection. To that end, we examined segments of aortic aneurysm wall, consisting of intima, media, and adventitia, collected from patients during aneurysm prosthetics intervention. The material was collected from 23 men and eight women aged from 33 to 69 years. Monoclonal antibodies to Lp(a), markers of monocytes and macrophages (CD68), T cells (CD3, CD4, and CD8), von Willebrand factor, endothelium NO synthase, and smooth muscle α-actin were used for morphological and morphometric investigation. The present study demonstrated that Lp(a) is not often found in biopsies of patients with thoracic aortic aneurysm. Morphological and morphometric investigation shows the connection of aortic dissection with the process of damage to its wall caused by inflammatory infiltrates, medianecroses, and the appearance of newly formed vasa vasorum in media.

## 1. Introduction

Aneurysms are defined as focal irreversible dilatations of all layers of the vessel wall that lead to an increase of the vessel diameter of at least one and a half times. Men suffer from this disease six times more often than women. Aneurysms of the thoracic aorta have different etiologies. While a monogenic etiology is characteristic of Marfan syndrome (mutation in the FBN1 gene encoding the fibrillin-1 protein) or Loeys-Dietz syndrome (mutations in TGFBR1, TGFBR2, and other genes involved in the transforming growth factor β (TGF-β) pathway), degenerative changes in the aorta are observed in older people. Patients with thoracic aortic aneurysm are often subject to such serious complications as aortic dissection and rupture. In the United States, the incidence of aortic dissection reaches 5–30 cases per one million people per year [1]. The total mortality rate for aortic rupture reaches 90%. It is believed that the following factors contribute to the rupture of the aorta: large diameter of the aneurysm (more than 6 cm) and the presence of hypertension. Choke suggested that the development of the delamination and subsequent rupture of the aortic aneurysm wall are largely due to the neovascularization of its wall and do not depend on the size of the inflammatory infiltrates [2].

Inflammatory infiltrates consist mainly of macrophages and T cells. Inflammatory infiltrates of the aortic aneurysm wall were found to contain CD4^+^ T cells, including Th1, Th2, and Treg cells and CD8^+^ T cells, including both CD8^+^CD27^−^ and CD8^+^CD28^−^ cells [3,4,5,6].

An important role in aortic wall damage is played by macrophages that are also present in the inflammatory infiltrates. Macrophages in the aneurysmal wall express matrix metalloproteinases (MMPs), which destroy the elastic and collagen fibers [7]. This leads to mechanical weakening of the aortic wall. The MMP family is closely involved in the process of neovascularization. MMPs play a key pro-angiogenic role, contributing to the formation of new vasa vasorum. The appearance of inflammatory infiltrates and newly formed vasa vasorum in the media of the aortic aneurysm can lead to the reduction of the aortic wall’s strength and subsequent rupture.

It was traditionally believed that abdominal aortic aneurysm has mainly atherosclerotic origin, while thoracic aortic aneurysm is more frequently associated with hereditary genetic diseases and dysplasia’s. The drawback of that model was that it did not take into account the role of lipoprotein (a) [Lp(a)] in the thoracic aortic aneurysm wall.

High level of Lp(a) is among the most common consequence of genetic dyslipidemia worldwide, affecting at least one in 5–10 people. Elevated Lp(a) is a risk factor contributing to myocardial infarction and aortic stenosis development [8]. The increase of the concentration of Lp(a) to ≥50 mg/dL leads to cardiovascular disease development. As soon as such a “pathogenic lipoprotein” enters the tissue, it binds to the extracellular matrix, contributing to the progression of the atherosclerotic process at the lesion site. Lp(a) is present in the arterial intima in both early and late stages of atherosclerosis. Lp(a) is present in calcified aortic valves. Subjects with elevated plasma Lp(a) levels are characterized by increased inflammatory activity of circulating monocytes [9]. Even a slight increase of Lp(a) concentration above the background can shift the contents of T lymphocyte subpopulations, increasing the risk of rapidly progressive and multi-vascular damage to the coronary arteries [9,10].

The aim of this work is to study the relationships among inflammation, the number of vasa vasorum, the presence of Lp(a), and the development of medianecrosis in the aortic aneurysm wall. We attempted to evaluate both the individual and combined impact of these processes on the development of thoracic aortic aneurysm dissection.

## 2. Materials and Methods

For this study, we collected segments of aortic aneurysm wall, consisting of intima, media, and adventitia. The material was obtained during the operation of aortic aneurysm prosthetics. We studied the biopsy material of the thoracic aorta of patients with a clinical diagnosis of thoracic aortic aneurysm. Human ascending thoracic aortic specimens (*n* = 47, collected from 31 patients) were collected during ascending aortic replacement operations. Informed consent was obtained from all patients. All patient-related procedures were carried out in accordance with the principles outlined in the Declaration of Helsinki. The length of aortic specimens was within 1–2 cm. In addition, we used archival material and extracts from case histories collected at the Russian Research Center of Surgery for a retrospective study. This work was approved by the local ethics committee.

Specimens were collected from 23 men and 8 women. The age of patients ranged from 33 to 69 years. Patients had the following comorbidities: 23 patients had arterial hypertension; 4 had coronary heart disease; 3 had chronic obstructive pulmonary disease; and 1 had myocardial infarction. Aortic segments taken from patients in the operating room were immediately fixed in 10% formalin and embedded in paraffin. All biopsies (47 paraffin blocks) obtained from 31 patients were stained with antibodies to Lp(a) (1:1000) (laboratory of Professor S.N. Pokrovsky, Federal State Budget organization National Medical Research Center of Cardiology, Russia) and to CD3 (Cat. N 05267293001 Roche, USA). Out of the 31 patients that provided samples for the study, eleven were studied in a blinded manner, without consulting their medical history. These biopsies were subjected to additional research using various antibodies. The study was divided into 2 stages. During the first stage, we performed assessment of inflammatory activity in the aneurysm wall and divided the test material into 3 groups. During the second stage, we assessed the density of vasa vasorum aorta in each group (11 patients). The immunohistochemical study was performed as follows: Ten serial sections with a thickness of 3 μm were made from each block. Serial slices were only “neighboring” slices (first and second; third and fourth, etc.), because the distance between them was either 0 or (maximum) 6 µm, and the distance between the first and last cut was 24 µm. The first slice was used as the staining control. From the second to the fifth slice, immunohistochemical (IHC) analysis with antibodies to CD3, CD4, and CD8 T cell antigens and CD68 macrophage antigen was performed. After that, according to the degree of inflammatory activity in the aortic aneurysm wall, all samples were divided into 3 groups (Group 1 with inflammatory activity in the aortic wall, Group 2 with single and small mononuclear cell infiltrates in adventitia, and Group 3 without inflammatory infiltrates). The second step was to compare the number of vasa vasorum in the aneurysm. For this, in each group, the sixth section was examined using antibodies to von Willebrand factor, and the seventh section was studied using antibodies to endothelial NO synthase. The IHC analysis of smooth muscle α-actin was performed on the eighth section. The ninth section was stained with hematoxylin and eosin; and the tenth section was spared. The IHC reaction was carried out on a Ventana immunohistostainer (Roche, USA). The monoclonal antibodies used in the study were the following: anti-CD68 (Cat. N 05278252001), anti-CD3 (Cat. N 05267293001), anti-CD4 (05552737001), anti-CD8 (Cat. N 05937248001), all from Roche, USA; anti-von Willebrand factor (1:800) (Cat. N F3520 Sigma -Aldrich, USA), anti-endothelium NO synthase (1:100) (Cat. N BF0705, Santa Cruz biotechnology, USA), anti-smooth muscle cell α-actin (Cat. N 05268303001, Roche, USA). After staining with antibodies to CD3, CD4, CD8, and CD68 antigens and α-actin, the preparations were stained with hematoxylin. Morphometric studies were performed using a ScanScope device from Leica, Germany, using the ImageScope morphometric program (Leicamycrosystems CMS Gmbh, Austria). The number of vasa vasorum was calculated over an area of ~ 500 μm^2^ (495 μm^2^ at a magnification of ×400) in six different, arbitrarily selected fields of view (in 6 fields of view in media and in 6 fields of view in adventitia) using the ScanScope instrument using the ImageScope morphometric program. In order to obtain the most objective data, all calculations and measurements were carried out by two researchers independently. Statistical analysis of the number of vasa vasorum was performed using the statistical analysis software Statistica 6.0. To assess the significance of differences in quantitative indicators, non-parametric Mann–Whitney U tests were used. Differences were considered significant at a confidence level of *p* ≤ 0.05.

## 3. Results

**Figure 1:**
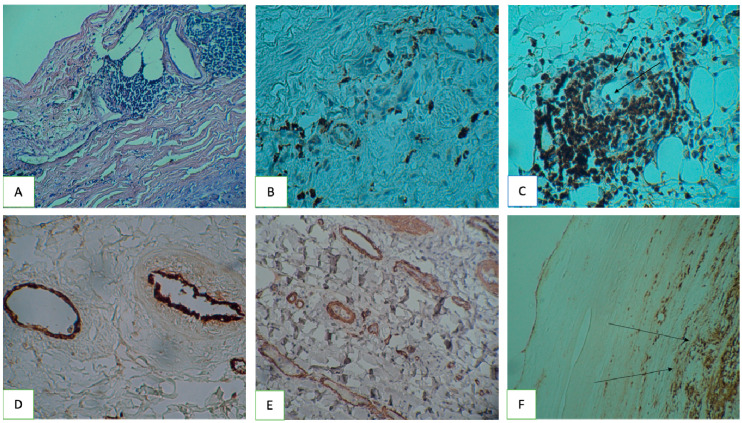
Inflammatory infiltrates and vasa vasorum in the aortic aneurysm wall, ICH staining. ICH—Immunohistochemistry (**A**) lymphoid infiltration of adventitial vasa vasorum. Hematoxylin and Eosin stain, magnification ×100; (**B**) CD4_+_ T cells in the area of the newly formed vasa vasorum, magnification ×200; (**C**) adherent CD8_+_ T-cells on the vasa vasorum endothelium (indicated by a long arrow), magnification ×200; (**D**) von Willebrand factor antibodies for detection vasa vasorum endothelium, magnification ×100; (**E**) vasa vasorum smooth muscle α-actin, magnification ×200; (**F**) Lp(a) accumulations in a fibrous plaque (indicated by a long arrow), magnification ×200.

At the first stage of the study, a histodiagnosis was performed. In the majority of cases, the intima of the aortic aneurysm was slightly thickened and sclerosed. In four cases, single small atherosclerotic (liposclerotic) plaques and fatty streaks were noted. In three cases, fibrotic plaques were found. In the medial layer of the aorta, the bundles of smooth muscle cells in some places had different directions and differed in thickness, and areas of connective tissue dysplasia were observed, sometimes (in seven patients), according to the type of cystic medianecrosis. In a number of cases (14 patients), revascularization of the middle membrane was noted. Fourteen patients had sections of arterial wall dissection along the middle layer with the development of common sclerotic changes in all layers. Histodiagnosis of six patients showed the presence of massive inflammatory infiltrates in the aortic aneurysm wall (Figure 1A). Inflammatory infiltration in these patients was not limited to adventitia, but expanded into media. In 11 cases, inflammatory infiltration of adventitia was moderate and did not spread to other areas. In the aortic aneurysm wall of 14 patients, inflammatory mononuclear cells could not be detected or were only detected as single cells in the field of view and were localized only in adventitia.

In total, forty-seven biopsy samples from 31 patients were examined using antibodies to Lp(a) and CD3. According to the inflammation degree of the aortic aneurysm wall, all samples were divided into three groups: Group 1: patients with high activity of the inflammatory process in the aneurysm wall; Group 2: patients with an insignificant degree of activity of the process; and Group 3: with no visible inflammatory infiltrates in the aortic aneurysm wall. In aortic aneurysm biopsies with a thickened intima, Lp(a) was detected in the form of clusters of granules, often merging with each other. These clusters did not extend beyond the basement membrane. In most samples from patients with aortic aneurysm, lipid spots did not contain Lp(a). In isolated cases (in three biopsy samples from two patients), it was possible to find the granules of the Lp(a), localized both in the center of lipid spots and on the periphery. In the thickening and scleroid intima, Lp(a) was found in the form of local granule accumulation (one patient). For one patient, liposclerotic plaques contained small granules of Lp(a). These granules were both on the periphery and in the center of plaques (see Figure 1E). Of the 47 biopsy specimens, Lp(a) deposits were detected in six cases (six patients). Single Lp(a) granules were found in 11 biopsy samples (11 patients). The comparison of biopsy samples showing Lp(a) deposits with those containing inflammatory infiltrates with CD3^+^ T cells did not reveal statistically significant differences (Table 1).

Biopsy samples of 11 patients with aortic aneurysm were immunomorphologically examined using antibodies to Lp(a) and CD3, as well as antibodies to CD4, CD8, and CD68 (see Figure 1B,C). Visually, CD4^+^ T cells and CD68^+^ macrophages dominated among the inflammatory mononuclear cells in the infiltrates. It is noteworthy that frequent localization of T cells and macrophages around vasa vasorum was observed. In places where inflammatory infiltrates surrounded vasa vasorum, T cells and CD68^+^ monocytes adhered to the endothelium could be seen in the vessel lumen (see Figure 1B,C; long arrows indicate CD8^+^ T cells adhered to the endothelium). According to the degree of inflammation in the aortic aneurysm wall, all samples were divided into three groups.

A morphometric count of vasa vasorum was performed in the same biopsy samples over an area of ~ 500 μm^2^ (or 0.5 mm^2^). Endothelium vasa vasorum was detected using antibodies to smooth muscle α-actin, von Willebrand factor, and NO synthase (Figure 1D,E). Previously, a relationship was shown between the activity of the inflammatory process in the aortic aneurysm wall and the number of vasa vasorum in its media. It was found that the density of newly formed vasa vasorum in the media of the aortic aneurysm in patients with an active inflammatory process was higher than in patients without active inflammation. This difference was statistically significant (*p* < 0.05).

Based on the results presented above, we hypothesized that the presence of aneurysm, inflammation, and a high density of vasa vasorum in the arterial wall weakens it mechanically, therefore leading to dissection. In our study, out of 11 examined patients, five had dissections. By comparing the studied material, we found that the number of dissections in the group of patients with extensive inflammatory infiltrates and a high density of vasa vasorum was greater than in the group of patients without these changes; however, this difference was not statistically significant.

In this study, the classification of patients into groups was based on of three parameters: (1) the presence of macrophage infiltrates in media, (2) vasa vasorum in media, and (3) aortic medianecrosis. We found that almost all patients with aortic dissection (four patients) had to be included in Group 1 (patients with active inflammation, high vasa vasorum density, and medianecrosis). In Group 2 (patients who did not have one or two of the listed symptoms), aortic wall dissection occurred in only one patient. The difference between these two groups was statistically significant (*p* < 0.05) (Table 2).

Thus, if in the aortic aneurysm biopsy of media macrophage infiltrates, a high density of vasa vasorum and of medianecroses were present, the risk of developing aortic dissection could be estimated as extremely high.

## 4. Discussion

In the research of van Dijk, where atherosclerosis in the coronary arteries of the heart was studied, the presence of Lp(a) in both early and late atherosclerotic plaques of the coronary arteries was found [11]. The present study demonstrated that Lp(a) was not commonly found in the biopsies of patients with thoracic aortic aneurysm. The inconsistency between our study and van Dijk’s results can be explained by different subjects that were studied and various pathological processes underlying these diseases [11]. Among the 47 aortic biopsies taken from 31 patients, Lp(a) was found only in six cases, wherein aortic dissection was absent in five of these six patients.

Biopsies with inflammatory infiltrates (in particular, from CD3^+^ T cells) rarely contained deposits of Lp(a). However, studies have shown that these observations are not statistically significant, i.e., the presence of active inflammation in the aneurysm wall does not exclude the deposition of Lp(a) in it.

Macrophages of inflammatory infiltrates express MMPs that cause disorganization of connective tissue and thereby weaken the strength of the aortic wall [7]. In a previous work, it was shown that patients with abundant inflammatory infiltrates in the aortic wall are characterized by an increased density of vasa vasorum in media [12]. Based on this, we suggested that these pathological changes in the aortic wall could contribute to its weakening, stratification, and rupture. In total, eleven randomly selected patients with aortic aneurysm were immunomorphologically examined, and morphometric measurements and statistical analysis of inflammatory infiltrates and vasa vasorum were performed. The results were compared with the amount of dissections in these patients. According to our data, the relationship between the presence of inflammatory infiltrates and the frequency of aortic dissection is not statistically significant. Choke came to the same conclusion [2]. A comparative analysis of aortic dissections and two studied parameters simultaneously (active inflammation of the aneurysm wall and the presence of vasa vasorum in media) also did not reveal a statistically significant relationship between them. Only the combination of three parameters (active inflammation of the wall of the aneurysm, the presence of vasa vasorum in media, and the appearance of medianecrosis) revealed a statistically significant relationship between these pathological changes and the development of the dissection of the aortic aneurysm wall. Thus, according to our data, if the biopsy of the aortic aneurysm in media is characterized by macrophage infiltrates, high density vasa vasorum, and medianecrosis, then the risk of developing aortic dissection is extremely high.

The importance of damage to the vasa vasorum of the aorta was also noted by other researchers. For instance, Osada examined samples of aortic aneurysm taken from 21 patients and concluded that damage to vasa vasorum leads to the development of medial necrosis and subsequent rupture of the aortic aneurysm [13]. Similar conclusions were reached by Angouras [14] and Tanaka [15]. They blocked the animals’ blood flow through the vasa vasorum, and this led to the rupture of the previously formed aortic aneurysm. The obtained data allowed them to conclude that vasa vasorum damage plays a major role in the development of aortic aneurysm dissection and rupture. Tanaka believes that damage to vasa vasorum leads to impaired blood flow through these vessels and the development of hypoxia in the aortic wall, which is complicated by inflammation and leads to damage to the aortic tissues [15]. Angouras and co-authors provided a slightly different explanation. They believed that damage to vasa vasorum causes increased stiffness of the outer wall of the aorta, because the diffusion of nutrients to it is insufficient. Therefore, the aorta, from a mechanical point of view, turns into a two-phase medium: internal and external. The inner part of the aorta receives sufficient nutrition and has normal elasticity, while the outer part (which receives nutrition from vasa vasorum) is ischemic and has increased stiffness. Shear stress occurs at the boundary of these media, which ultimately leads to aortic dissection [14].

## 5. Conclusions

Our working hypothesis for the present study was that damage to vasa vasorum leads to a violation of blood supply to the outer part of the aortic wall. Chronic ischemia of a part of the aortic wall (in particular, the outer 1/3 of media) causes the development of media necrosis, damage to the elastic and collagen fibers, and ultimately, dissection and rupture of the aortic wall with possible fatal consequences. In order to compensate for chronic ischemia of the aneurysm wall, imperfect newly formed vasa vasorum appear in media, as a response to with hypoxia and impaired aortic aneurysm wall nutrition. If the appearance of such newly formed vasa vasorum appears to be insufficient to improve the oxygen and nutrient supply, new medianecrosis occurs in the aneurysm wall, and the old lesions are not repaired. This is exactly what we found in patients with aortic dissection: (1) the appearance of newly formed vasa vasorum in media, (2) the presence of medianecroses in the aneurysm wall, and (3) the presence of CD68^+^ macrophage infiltrates.

## Figures and Tables

**Table 1:** Aortic aneurysm biopsy. Comparison of inflammatory infiltrates from T cells with Lp(a) deposits.

Number of T Cells	Lp(a) (-)	Lp(a) (++)	Lp(a) (+++)	P1,2	P1,3	P2,3
CD3 (-)	14	7	4	1.0	0.6581	0.4841
CD3 (++)	10	2	0	0.5147	0.1567	0.4566
CD3 (+++)	6	2	2	0.584	0.5963	1.0

P1,2: statistical difference between Groups 1 and 2. P1,3: statistical difference between Groups 1 and 3. P2,3: statistical difference between Groups 2 and 3.

**Table 2:** Patients with aortic aneurysm, morphological characteristics of the wall.

No.	Vasa Vasorum in Media: The Average Number Per Unit Area *	CD68	Medianecrosis	Dissection
1	2.3	+	+	+
2	2.6	+	+	+
3	1	+	+	+
4	2.6	+	+	+
5	0	-	+	-
6	0	-	+	-
7	0	-	-	-
8	0	-	+	+
9	0	+	-	-
10	0	+	-	-
11	0.2	+	-	-
Sensitivity	0.71	0.57	0.77	0.8 (counting all 3 parameters)
Specificity	0.83	0.5	0.66	0.85 (counting all 3 parameters)

* Area of 500 μm^2^.
